# A feasibility and usability study of a virtual reality tool (VESPA 2.0) for cognitive rehabilitation in patients with mild cognitive impairment: an ecological approach

**DOI:** 10.3389/fpsyg.2024.1402894

**Published:** 2024-10-18

**Authors:** Desirèe Latella, Caterina Formica, Augusto Ielo, Pietro Grioli, Angela Marra, Daniela Costanzo, Maria Emanuele Merlo, Salvatore Marco Pappalardo, Francesco Corallo, Silvia Marino, Angelo Quartarone, Rocco Salvatore Calabrò, Giuseppa Maresca

**Affiliations:** ^1^IRCCS Centro Neurolesi “Bonino-Pulejo”, Messina, Italy; ^2^Department of Biomedical and Dental Sciences and Morfofunctional Imaging, University of Messina, Messina, Italy; ^3^Software Engineering Italia S.r.l., Via Santa Sofia, Catania, Italy

**Keywords:** mild cognitive impairment, virtual reality, neurological disorder, cognitive rehabilitation, neurorehabiliation

## Abstract

**Introduction:**

Mild cognitive impairment (MCI) or “mild neurocognitive disorder” represents an intermediate status between normality and dementia. It is characterized by cognitive decline that does not significantly interfere with normal daily living activities. Virtual reality (VR) is the new frontier of rehabilitation.

**Methods:**

We enrolled 50 MCI patients who underwent a neuropsychological evaluation and participated in 40 sessions of cognitive treatment using the Virtual Environment for a Superior Neuro-Psychiatry, Second Generation (VESPA 2.0) System. This preliminary study highlights the role of VR tools for cognitive rehabilitation (CR) for the recovery of cognitive functions and consequent better management of MCI condition. Our study demonstrated that the VESPA 2.0 System is a valuable tool in a context that closely resembles real-life situations rather than controlled, artificial environments as traditional cognitive training methods.

**Results:**

The results showed that the patient group had significant improvements between T0 and T1 (assessment), in particular, in the global cognitive profile, visuospatial skills, and executive functions after treatment with the VESPA 2.0 System.

**Discussion:**

Our findings contribute with new evidence of understanding the impact of using simulations of the Activities of Daily Living (ADL) scale in the CR.

## 1 Introduction

Mild cognitive impairment (MCI) is a condition intermediate between normal and dementia and refers to cognitive decline that does not interfere with normal daily activities (Anderson, 2019; Jongsiriyanyong and Limpawattana, 2018; McKhann et al., 2011; Langa and Levine, 2014). The classification and misdiagnosis of MCI in this population have been a challenge for the scientific community. One important issue is the potential for contamination of MCI diagnoses in healthy individuals. Indeed, some MCI patients may have impaired memory function that does not progress. However, the proportion of MCI patients who have had long-term memory decline is too small to be objective due to the lack of longitudinal data. Some of these patients could be classified as MCI (Petersen et al., 1999). A second important issue regarding the difficulty of classifying MCI concerns the use of instruments that are not linear or are less sensitive to changes in milder conditions. As noted earlier, the MCI group may be “contaminated” with essentially healthy subjects who do not progress to Alzheimer’s disease (AD). The proportion of individuals with MCI who progress to AD ranges from 10 to 15% per year (Okello et al., 2009). The MCI incidence rates are 6.7% for ages 60–64, 8.4% for ages 65–-69, and 10.1% for ages 70–74, 25.2% for ages 80-84. The cumulative incidence of dementia in patients with MCI aged 65 years and older followed for 2 years was 14.9%. There is no high-quality evidence to support pharmacological treatment for MCI. In patients with MCI, physical and cognitive exercise training (6 months) may improve cognitive function (Petersen et al., 2018). MCI can be classified as amnestic or non-amnestic and can be divided into four subtypes: (i) single-domain amnestic if only the memory domain is impaired, (ii) single-domain non-amnestic if the memory domain is not impaired but single cognitive domain is impaired, (iii) multiple-domain amnestic if memory and other cognitive domain showed impairment, and (iv) multiple-domain non-amnestic if the memory domain is not impaired and other cognitive domains are impaired. The subtypes of multiple-domain amnestic MCI and single-domain non-amnestic MCI were significantly associated with the development of dementia for both diagnoses; in particular, AD was associated with a diagnosis of single-domain amnestic and non-amnestic MCI (Jak et al., 2016; McCarten, 2013); non-amnestic cognitive decline is relatively less common and is often more difficult to diagnose (McCarten, 2013; Holsinger et al., 2007). The lack of a universally accepted approach to the objective identification of cognitive impairment and a wide range of conceptual and diagnostic approaches to MCI have led to highly variable prevalence rates from 1 to 30% (Cabeza et al., 2018). However, other studies have employed larger neuropsychological test batteries that formally assessed multiple cognitive domains, demonstrating the importance of using comprehensive neuropsychological assessments as an essential variable to define subtype and level of gravity (Sherman et al., 2017; Liao et al., 2020). Another important issue is the prediction of MCI progression in individuals, and the clinician’s main goal should be early screening and pre-diagnosis since it represents a major risk factor that can be identified and treated to prevent or delay potential progression to dementia (Plaza-Zabala et al., 2017). One study on multi-comorbidity and the development of MCI found that patients with four or more chronic diseases, particularly hypertension, hyperlipidemia, coronary artery disease, and osteoarthritis, were at highest risk for MCI (Winblad et al., 2004). Lifestyle also plays an important role in the risk of developing MCI, including a sedentary lifestyle and lack of physical exercise (Vassilaki et al., 2015; Kim et al., 2022). The need for early screening and diagnosis is useful to better manage this condition (Ren et al., 2024). Management of clinical risk factors (e.g., hypertension, atrial fibrillation, and diabetes) is essential to prevent ischemic damage and slow the progression of cognitive decline. Non-pharmacological interventions are crucial in empowering individuals to manage their condition actively and achieve better long-term outcomes. For this reason, much attention has focused on cognitive and exercise rehabilitation and maintenance of a healthy lifestyle as protective factors. In 2022, Kim et al. analyzed the therapeutic effects of VR resulting in positive effects on cognitive function in individuals with MCI. However, there was no significant improvement in the subcategories such as global status cognition, executive function, working memory, memory functioning, and attention. Previous results differed from those reported by previous meta-analyses (Yu et al., 2022; Cherniack, 2011), which showed significant improvements in global cognition. The overall results were not consistent with those from previous reviews. This discrepancy might have been due to differences in methodological factors and analyses. A systematic review by Yu et al., Geda et al. (2010) found that semi-immersive and non-immersive VR types are more effective than immersive VR as immersive technologies can be complex and challenging for individuals with MCI (Roberts et al., 2014). Available rehabilitation therapies for MCI focus on motivation and social participation. Cognitive rehabilitation (CR) therapy is an evidence-based intervention to stimulate and engage patients with MCI because it promotes social participation, learning, and recall and improves participants’ cognition and quality of life (Eshkoor et al., 2015; Campbell et al., 2013; Livingston et al., 2020). CR training varies depending on the patient’s needs and characteristics. It includes cognitive exercises and training (e.g., puzzles, memory games, and attentional exercises), compensation strategies (e.g., using memory aids/organizing daily routines), and psychoeducation and counseling for the patient/their caregivers. CR assumes that interaction with the external environment enhances neuroplasticity (Eshkoor et al., 2015). Although previous studies have highlighted the beneficial role of CR, from traditional paper-and-pencil methods to more innovative tools such as PC-based ones, these interventions showed many limitations, including restrained accessibility, financial constraints, and geographical barriers. To overcome these challenges, new techniques have been applied, and virtual reality (VR) offers a promising alternative through a series of informatics technologies by creating immersive and interactive environments that simulate real-life scenarios and engage the user (Ahlskog et al., 2011; Tortora et al., 2023; Lasaponara et al., 2021). These systems consist of specific software programs and input/output peripherals that recreate complex and immersive experiences; VR systems can adjust the difficulty of activities according to the patient’s abilities and potential. The system can be used to control performance through visual and auditory feedback. In addition, these systems improve the quality of rehabilitation sessions by offering the opportunity to propose playful activities, increasing motivation and participation. VR rehabilitation improves impaired function in a variety of neurological disorders (Ahlskog et al., 2011; Tortora et al., 2023), stimulates and improves residual abilities, and promotes psychological wellbeing, participation, and autonomy. Patients perform exercises while interacting with virtual scenarios and audio-visual stimuli, resulting in full engagement of the sensory system and improvements in specific cognitive domains such as attention, memory, language, executive function, spatial cognition, perceptual abilities, and anxiety.

### 1.1 Levels of VR immersion

VR can be categorized based on the level of immersion it offers: Non-immersive VR involves a computer screen or a projection display where users view a virtual environment and interact using input devices such as keyboards, mice, or gamepads. Non-immersive VR is cost-effective and easily integrated into apps for various devices such as smartphones or tablets, and its lower level of immersion may limit its ecological validity; semi-immersive VR offers a higher level of immersion. It involves larger or multiple display screens, providing a wider field of view to patients. Specialized glasses and haptic feedback or motion tracking enhance the virtual experience. Semi-immersive VR is the right balance between immersion and practicality but may still face some limitations in realism and the patient’s engagement; fully immersive VR creates a complete sense of presence within the virtual environment. It uses head-mounted displays that cover the user’s eyes and ears, blocking out the real world and replacing it with a virtual one. Motion tracking technology detects the user’s movements and adjusts the virtual environment, accordingly, allowing users to freely explore and interact. Fully immersive VR offers the most realistic experience but is often more expensive and can cause motion sickness or nausea due to sensory conflicts. Each VR system has specific advantages and disadvantages. Non-immersive VR is more accessible and cost-effective but less immersive. Semi-immersive and fully immersive systems provide more realistic experiences but come with higher costs and potential hardware limitations. These limitations can affect their reach and accessibility, particularly among older adults who may not have access to VR-ready devices. Therefore, the choice of immersion level should be based on individual characteristics and needs (Wu et al., 2020). In general, VR-based healthcare offers numerous benefits, particularly for individuals with mobility and economic challenges. VR allows patients to access rehabilitation and training programs regardless of their location, reducing the need for travel and minimizing waiting times. VR cognitive rehabilitation can be conducted remotely, enabling individuals to receive rehabilitation services from their own homes (Catania et al., 2024). In addition, VR systems can adapt task difficulties in real time based on an individual’s performance, making cognitive rehabilitation more engaging, motivating, and beneficial (Cabeza et al., 2018). This approach is considered more ecologically valid than conventional cognitive rehabilitation therapy (CRT) because VR replicates real-world contexts and situations, helping individuals transfer skills and strategies acquired in the virtual environment to daily life.

### 1.2 Ecological validity in virtual environments

Human functioning is best understood within its natural environment, where interactions are complex and active. Laboratory studies, while useful, may sometimes fail to provide a complete picture due to their often limited ecological validity. This is particularly significant in behavioral neuroscience, where understanding natural behavior is crucial for identifying the true neural mechanisms involved. VR allows for a high degree of experimental control while simulating real-life environments closely (Sherman et al., 2017). VR provides controlled and repetitive exposure to stimuli and tasks, facilitating targeted cognitive training. Although VR environments can be tailored to simulate real-world scenarios and challenges, these simulations do not reproduce the unpredictability and complexity of everyday life. Clinical research must find a balance between controlled training environments and ecologically valid scenarios to ensure that goals achieved in VR are applicable effectively into the world. Moreover, assessing the ecological validity of VR-based cognitive rehabilitation necessitates a multifaceted approach. It involves not only the virtual environment but also the individual’s ability to generalize skills and strategies learned in VR to real-world settings. VR long-term studies are crucial for evaluating ecological validity. The interdisciplinary team is decisive in fine-tuning VR applications, ensuring the complexities of real-life challenges and the achievement of rehabilitation goals.

### 1.3 VESPA system

The VESPA 2.0 project is a research project that aims to diagnose and rehabilitate cognitive-motor function in patients with intellectual disabilities (Liao et al., 2020) by developing and validating an immersive 3D VR computerized system that can be remotely monitored by a remote medical professional. The aim is to validate the effectiveness of individualized focused cognitive stimulation (SCI-i) interventions for general and specific cognitive functions in patients with mild or moderate cognitive decline. The VESPA System is a device connected to a European-scale Cloud Computing network for the assessment and rehabilitation of cognitive and motor functions through high-immersion virtual reality and supervision via videoconference (supervision) by specialized personnel. Compared to other projects, it goes beyond the local and unique vision of the service, to create a Europe-wide network. The VESPA 2.0 System expands assessment and rehabilitation capabilities by introducing new diagnostic and rehabilitative software, creating a network of virtual reality environments (Virtual Rooms—CV—of the type CAVE)^1^ evolved that surpass the concept of 3D by implementing sensory stimulation (olfactory, tactile, etc.) and dynamic interaction (e.g., assisted ambulation). In particular, the system will be based on the high-performance reality (HPR) solution, which combines supercomputing with extended reality (XR). This concept envisions the “structured and disciplined” coexistence and interaction of high-performance computing (HPC) systems and high-performance visualization (HPV) systems within extended reality. HPR represents a new tool for data analysis and related evaluations, enhancing the power of decision-making processes while simultaneously reducing the time required. The data (and models) derived from calculation, detection, and modeling sessions, processed by the HPC component, serve as the input for the immersive and interactive visualization system. This system allows for the evaluation and visual analysis of the data in an entirely innovative manner, leading to unprecedented optimization of evaluative and decision-making processes. Patients eligible must have cognitive-motor deficits and can benefit from quantitative assessment and rehabilitation of cognitive and motor functions. In immersive virtual reality, the individual manipulates virtual objects experiencing tactile sensations, while in the distributed system (SD) of VESPA 2.0, the patients move well beyond the space of virtual chambers (CVs) and experience olfactory, auditory, etc., sensations for deeper neurological stimulation and a broader evaluation of visual–spatial deficits (perceptual, attentional, and mnemonic). The network of distributed virtual chambers constituting the distributed virtual space connects various sites, creating in Sicily the first network of Rehabilitation Centers in SD in the world. Some modules of the VESPA 2.0 System will be included in the home version, which will use a tablet, head-mounted display, and Microsoft Kinect (or similar devices). The results will be transmitted and processed in real time via the Internet to immediately reconfigure the rehabilitation protocol. The centralized integration of virtual chamber results reduces costs for managing individual terminals, improves performance, and facilitates data collection for epidemiological and scientific research purposes. The centralized integration of CV results in the rehabilitation system not only reduces costs related to the management of individual terminals but also improves the effectiveness and efficiency of treatment, offering significant benefits to both patients and healthcare providers. In particular, each rehabilitation center does not need to own and maintain expensive local infrastructure. Technological resources, such as servers and data processing systems, are shared at a regional or national level, distributing costs over a larger number of users. The use of tablets, head-mounted displays, and Microsoft Kinect (or similar) can be optimized through centralized integration, reducing the need for multiple purchases and frequent updates for each individual center. Specialists can monitor and assist patients remotely, reducing the need for on-site staff and allowing for more efficient use of human resources. It facilitates the training of staff, allowing the sharing of best practices and the most effective rehabilitation protocols among different centers. The real-time transmission and processing of results allow for immediate adaptation of rehabilitation protocols and the customization of treatments based on the specific needs of patients improving treatment effectiveness. Moreover, the maintenance of equipment and software can be managed centrally, reducing maintenance and update costs for each individual center. The VESPA 2.0 project introduces also the generation and analysis of Big Data, integration of support for the health record, secure teletransmission, and advanced privacy protection of patient data. In this study, we used a tablet-based, home-based module. This preliminary study is part of the larger VESPA 2.0 research initiative, which explores the use of VR tools for cognitive rehabilitation (CR) in individuals with mild cognitive impairment (MCI). Specifically, this preliminary study aims to highlight the rehabilitative potential of VR tools (VESPA 2.0) for cognitive rehabilitation (CR) in individuals with mild cognitive impairment (MCI). The focus is to demonstrate how a targeted cognitive rehabilitation process using VR can lead to the recovery of cognitive functions, thereby improving the management of the MCI condition.

## 2 Materials and methods

### 2.1 Study population

We employed purposive sampling to select participants for this study. Purposive sampling is a non-probability sampling technique used when specific criteria are required to ensure the sample aligns with the research objectives. Purposive sampling was chosen to ensure that all participants had similar characteristics relevant to the study’s aims, particularly the diagnosis of MCI. This method allowed us to focus on a specific subgroup of the population to better understand the effects of VR on cognitive function in individuals with MCI. Fifty patients (mean ± SD age: 69.3 ± 6.5 years; 50.0% male) were admitted to the Neurorehabilitation Unit of the IRCCS Centro Neurolesi Bonino Pulejo—Piemonte (Messina, Italy). The training was conducted in 5 months between October 2022 and March 2023. Participants were enrolled by the clinicians and evaluated by a neuropsychologist before the treatment. The inclusion criteria for participants were as follows: (i) mild cognitive impairment; Montreal Cognitive Assessment (MoCA) scores under 22; CDR score 0.5, (ii) age > 55 years old, (iii) sign informed consent (iii). The exclusion criteria were as follows: (i) comorbidities with psychiatric syndromes, (ii) informed consent not signed, (iii) typical parkinsonism, (iv) clinical conditions involving problems related to vision and language. This study was conducted in accordance with the Declaration of Helsinki 1964 and approved by the Local Ethics Committee of IRCCS Centro Neurolesi “Bonino Pulejo”(protocol code 47/21 approved on 6 October 2022), and written informed consent was obtained from all participants. The data are available.^2^

### 2.2 Clinical assessment

Each participant was assessed by a neuropsychological evaluation before (T0) and immediately after the end of the cognitive training (T1). The evaluation was conducted blind by a neuropsychologist to ensure that cognitive assessments and interpretations of results remain unbiased and objective. This is crucial even in studies without a control group as it prevents any unconscious influence on the evaluation process that could arise from knowing the treatment details. Although it is ideal to include a control group to maximize the internal validity of the study, there are situations where this is not feasible. In our case, two practical limitations were presented: The population available for the study was limited, due to the post-COVID pandemic effects, and the European Commission has published a study on the impact of the EU’s policies on the environment. Actual neuropsychological criteria have expanding support in the literature for improving diagnostic rigor for MCI (Nasreddine et al., 2005). An objective neuropsychological impairment in the classification of MCI is the most variable and ill-defined component of the diagnosis (Raven, 2000). For this reason, we considered to use different neuropsychological tests to analyse specific cognitive domains to obtain a more complete neuropsychologic profile. Our assessment included a global cognitive evaluation using the following: Montreal Cognitive Assessment (MoCA; Tommasini et al., 2022) was designed as a rapid screening instrument for mild cognitive dysfunction. It assesses different cognitive domains: attention and concentration, executive functions, memory, language, visuospatial skills, conceptual reasoning, and orientation. Based on the MoCA outcome, we proceeded to use tests to assess specific cognitive domains as follows: Standard Progressive Matrices (SPM) which measures logical reasoning and visuospatial organization (Dubois et al., 2000); the Rey-Osterrieth Complex Figure Test (ROCFT; Lawton and Brody, 1969) to assess visuospatial memory; Frontal Assessment Battery (FAB; Borsci et al., 2009) to examine executive functions such as categorization, cognitive flexibility, and inhibition. Finally, we assessed autonomies in the daily activity functioning with the Activities of Daily Living (ADL) scale and Instrumental ADL (IADL; Maggio et al., 2018). At the end of the rehabilitative training, we used a scale for evaluating the main dimensions of usability by using an Italian version of the System Usability Scale (SUS; Manuli et al., 2020). The neuropsychological battery is described in Table 1.

**Table 1:** Neuropsychological assessment.

Test	Cognitive domains investigated	Number of items	Scoring range	Psychometric properties
Montreal Cognitive Assessment (MoCA)	Multiple cognitive domains: attention, memory, language, visuospatial skills, executive functions, calculation, and orientation	30	0–30	High reliability and validity for detecting mild cognitive impairment; sensitivity: 90%, specificity: 87%. High test–retest (0.945) and inter-rater (0.999) reliability. Sensitivity and specificity are adequate at 95.3 and 84.5%, respectively.
Visual Search	Attention and visual scanning	Varies (typically 60–120)	Number of correct responses and time to completion	Test–retest reliability (*r* = 0.80); generally used to assess visual attention and processing speed
Complex Figure of Rey	Visuospatial abilities, memory, planning, and organization	N/A	Copy accuracy and recall score (typically 0–36)	Reliability (*r* = >0.90). Pearson correlations: accuracy scores for copy and recall is *r* = 0.93 and recall *r* = 0.97
Activities of Daily Living (ADL)	Basic daily activities: bathing, dressing, eating, etc.	Varies (typically 6)	Independence level (score varies by tool)	Measures functional status; high reliability and validity; commonly used in clinical and research settings. Good test–retest reliability (0.41–0.70) and high inter-rater reliability (0.85)
Instrumental Activities of Daily Living (IADL)	More complex daily activities: using the phone, managing finances, and medication management	Varies (typically 8)	Independence level (score varies by tool)	Assesses more complex aspects of daily living; high reliability and validity in older adult populations. Good test–retest reliability (0.41–0.70) and high inter-rater reliability (0.85)
System Usability Scale (SUS)	Usability of a system tool	10	0–100 (converted to percentile rank)	Widely used tool for assessing usability; high reliability (*r* = 0.822); validated across various systems

### 2.3 Study design

All patients, before starting the cognitive treatment, were admitted to a neuropsychological unit at baseline (T0). In a previous session, they were trained to use the DEMO version of the VESPA 2.0 Tablet; then, they carried out a total of 40 sessions of cognitive rehabilitation. Each session was performed for 45 min 2 times a week for a total of 40 sessions. The training was conducted in 5 months (from October 2022 to March 2023). After this period, patients were evaluated with neuropsychological assessment (T1). VESPA 2.0 is based on an integrative and ecological approach used for the treatment of cognitive dysfunctions in patients with MCI or other neurodegenerative disorders. In our preliminary study, we used a DEMO version on a tablet touch screen of the 3D room with the same exercises. On the home page of the tablet, the patients saw all the tasks divided into three sections: AD for daily activities (see Figures 3A,C; Table 2); COG for logical deductive reasoning (see Figure 3B; Table 3); and ADK for visuospatial abilities (Figure 3D) in which patients performed exercises in which patients have to rearrange cubes (according to number or letter sequences) or remember a visual–spatial formation and then reproduce it (Table 4). Each section included five tasks of 10 min each. Every task in the VESPA program varies in difficulty levels. Patients were induced by the operators to select the section that deals with the cognitive domain in which the patients showed the most deficits. The patients were always followed by an operator during each treatment. Each task was preceded by instructions that appeared on the screen and were also given verbally by an automatic voice, and with a help button, the patient will be able to recall the instructions of the activity. Every activity was conducted with the touch screen modality, and the system produced visual and auditory feedback to signal the error or the achievement of the objective. After a series of errors, the training proceeds with helping cues until the correct performance is achieved. In this study, a control condition was not included.

**Figure 1:**
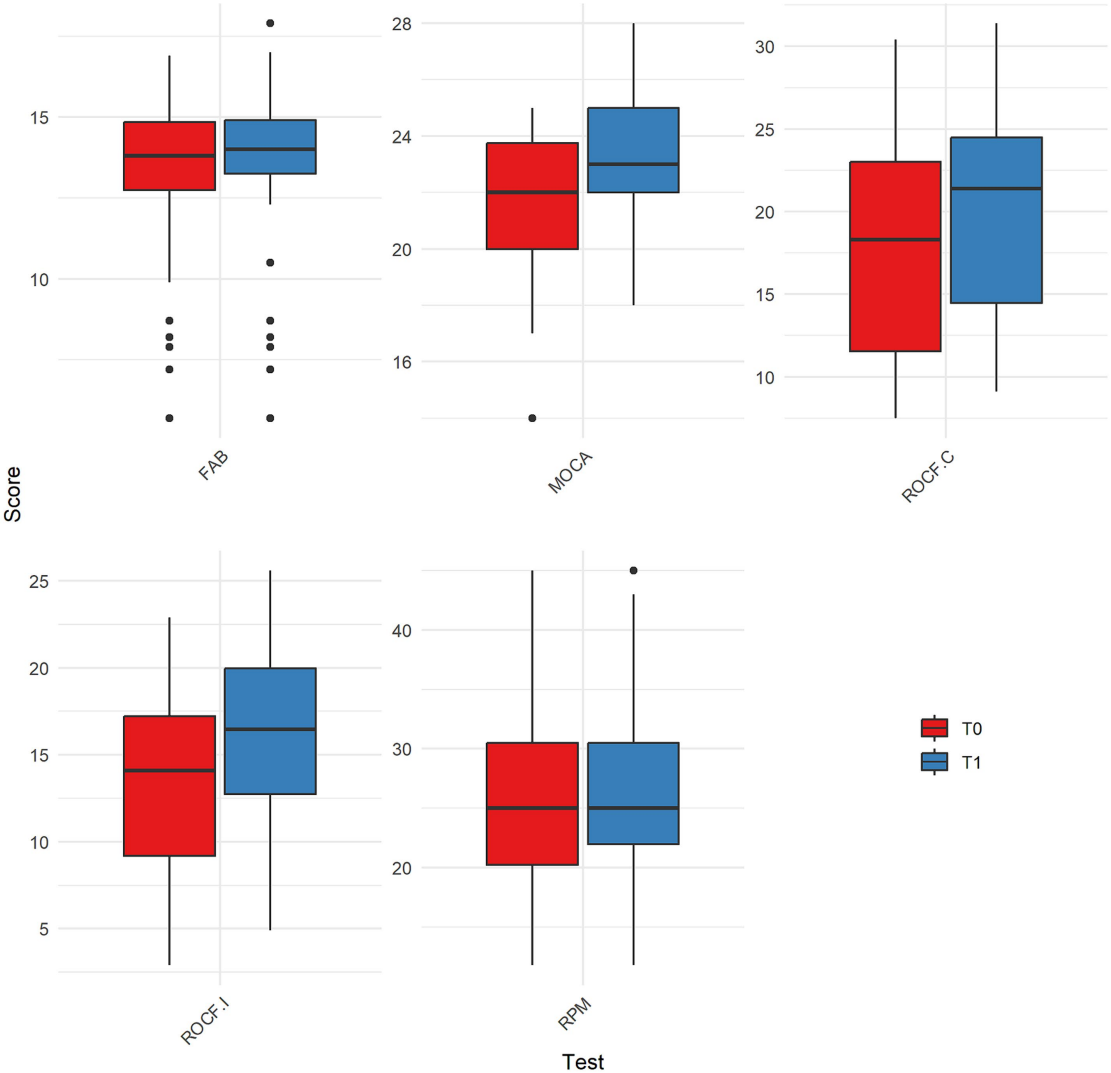
Comparison of scores between T0 and T1 for significantly changed clinical assessments. MOCA = Montreal Cognitive Assessment; RPM = Raven’s Progressive Matrices; ROCF.C = Rey–Osterrieth Complex Figure – Copy condition; ROCF.I = Rey–Osterrieth Complex Figure – Immediate recall condition; FAB = Frontal Assessment Battery; T0 = baseline; T1 = follow-up.

**Table 2:** Description of the AD domain tasks.

Task name	Activity description
AD 1	Prepare an orange juice for a friend
AD 2	Recall the sequence of actions previously done to prepare the juice
AD 3	Pack a suitcase for a vacation in Rome
AD 4	Recall the sequence of actions previously done to pack the suitcase
AD 5	Personal orientation (name, surname, age)
AD 6	Spatial orientation (where are you? In which city?)
AD 7	Temporal orientation (what is the date today?)

**Table 3:** Description of the COG domain tasks.

Task name	Activity description
COG 1	Arrange even and odd numbers
COG 2	Sort the cubes by size
COG 3	Arrange numbers in sequence (1-2-3-...)
COG 4	Sort the plants by type
COG 5	Sort the fruit trees (lemons and oranges)
COG 6	Collect the fruits from the tree and place them in baskets
COG 7	Arrange the race cars by their number (even and odd)
COG 8	Sort the clothes by category
COG 9	Arrange the groceries inside the refrigerator
COG 10	Do the recycling
COG 11	Arrange the cubes by color
COG 12	Arrange the players on a soccer field
COG 13	Make the bed
COG 14	Catch the fish based on their color
COG 15	Sort the playing cards by suit
COG 16	Play Klondike

**Table 4:** Description of the ADK domain tasks.

Task name	Activity description
ADK 2	Reproduce the sequence of cubes as per the model based on the position
ADK 4	Reproduce the sequence of cubes as per the model based on the position after memorizing it
ADK 6	Identify the cube in the direction indicated by the arrow
ADK 8	Arrange the cubes by pairing letters and numbers (e.g., 1A-2B)

### 2.4 Statistical analysis

The data were analyzed using R version 4.2.2, considering a *p*-value of <0.05 as statistically significant. The Shapiro–Wilk test was applied to assess the normality of the distributions of the variables at T0 and T1. A non-parametric analysis was carried out. The Wilcoxon signed-rank test was used to compare the scores between baseline and follow-up (intra-group analysis; Figure 1).

**Figure 2:**
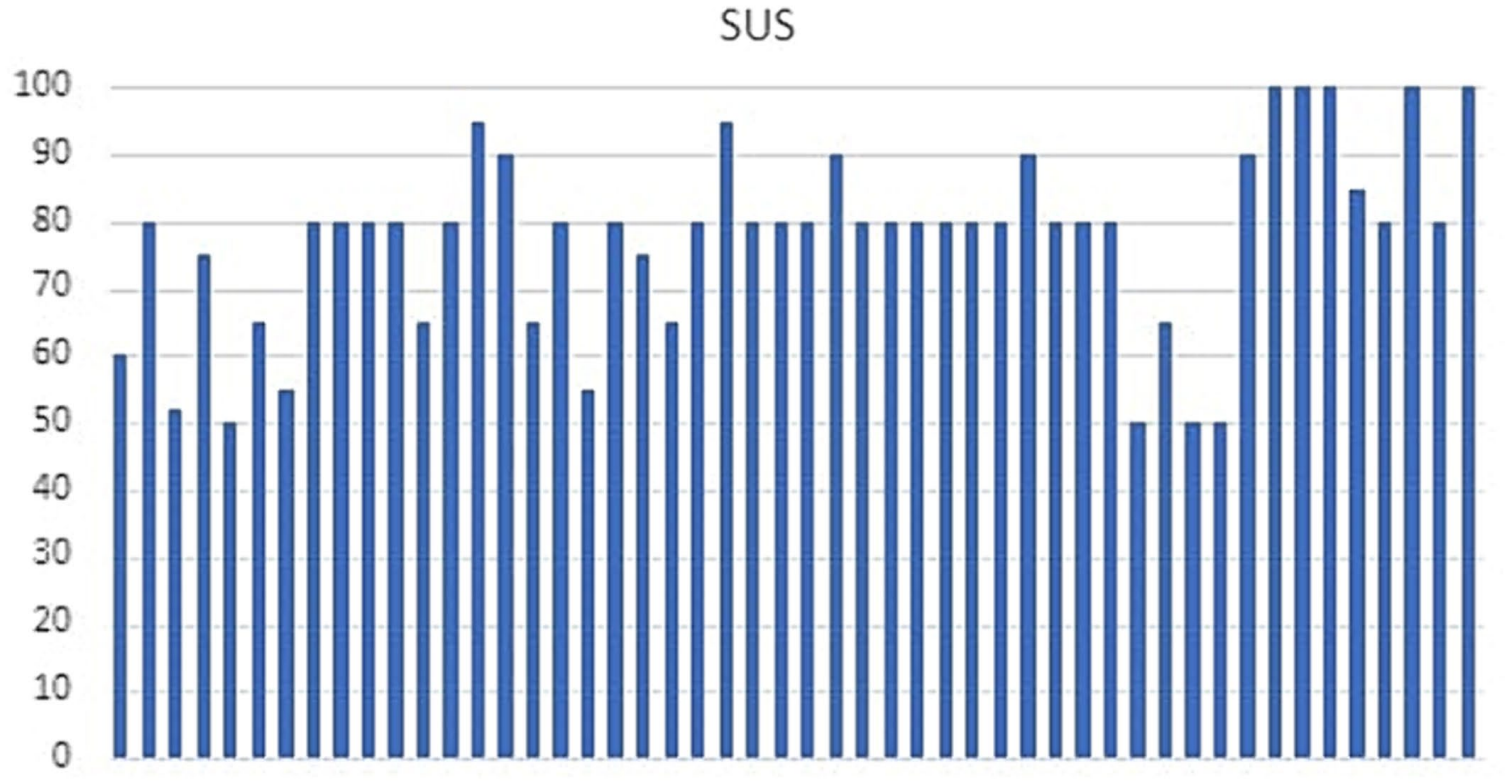
System Usability Scale (SUS) scores.

## 3 Results

The socio-demographic details of the sample are described in Table 5. The Shapiro–Wilk test revealed a significant deviation from normality; therefore, a non-parametric analysis was performed. The results of the statistical analysis are shown in Table 6. Improvements from T0 to T1 in cognitive assessment were found. Specifically, MOCA scores showed a significant increase, with median scores rising from 22 at T0 (IQR = 3.8) to 23 at T1 (IQR = 3). The Wilcoxon signed-rank test confirmed this change as statistically significant (*Z* = −5.30, *p* < 0.001). Similarly, RPM scores showed significant improvements, with median scores increasing from 25 at T0 (IQR = 10.2) to 25 at T1 (IQR = 8.5). The statistical analysis revealed this improvement to be significant (*Z* = −2.70, *p* = 0.009), suggesting enhanced cognitive abilities post-intervention. Furthermore, FAB scores increased from a median of 13.8 at T0 (IQR = 2.1) to 14 at T1 (IQR = 1.6), with a significant result (*Z* = −2.80, *p* = 0.005), reflecting improvements in executive functions. The ROCFT copy condition also saw significant enhancement, with median scores improving from 18.3 at T0 (IQR = 11.4) to 21.4 at T1 (IQR = 10.0). This change was statistically significant (*Z* = −5.80, *p* < 0.001), indicating better visuospatial memory abilities. Moreover, the immediate recall condition of the ROCFT revealed a significant improvement (*Z* = −5.70, *p* < 0.001), with median scores rising from 14.1 at T0 (IQR = 8.0) to 16.5 at T1 (IQR = 7.2) for cognitive assessment: MOCA (*p* < 0.001), RPM (*p* = 0.009), FAB (*p* = 0.005), ROCF copy Figure 1. No significant differences between T0 and T1 scores were found for the ROCFT delayed recall condition (*p* = 0.371), While ADL and IADL scores were equal between T0 and T1 for all subjects. The usability of the VESPA 2.0 system was evaluated with SUS (M = 77.44; SD = 14.11) demonstrating a good level of facility to use for all samples (Figure 2). Of the 50 patients, 13 scored a SUS below 68 (26%) and 37 above 68 (74%).

**Table 5:** Description of socio-demographic details of sample.

N. of subjects	50
**Gender**	
Male	25 (50%)
Female	25 (50%)
**Age**	
59–69	24 (48%)
69–79	23 (46%)
79+	3 (6%)
Mean **±** SD	69.3 ± 6.5
**Education**	
Secondary school	24 (48%)
High school	16 (32%)
Undergraduate / postgraduate	10 (20%)
**Status**	
Married/cohabiting	46 (92%)
Widowed	4 (8%)

Continuous variables were expressed as mean ± standard deviation, whereas categorical variables as frequencies (percentages).

**Table 6:** Statistical comparisons of clinical scores between baseline (T0) and follow-up (T1).

Clinical assessment	T0	T1	*p*-value
MOCA	22.0 (20.0–23.8)	23.0 (22.0–25.0)	< 0.001
RPM	25.0 (20.3–30.5)	25.0 (22.0–30.5)	0.009
ROCF.C	18.3 (11.6–23.0)	21.4 (14.5–24.5)	< 0.001
ROCF.I	14.1 (9.2–17.2)	16.5 (12.8–20.0)	< 0.001
ROCF.D	10.7 (5.2–16.5)	10.7 (5.2–19.6)	0.371
FAB	13.8 (12.8–14.9)	14.0 (13.3–14.9)	0.005
ADL	5.0 (5.0–5.0)	5.0 (5.0–5.0)	NA
IADL	6.5 (5.0–8.0)	6.5 (5.0–8.0)	NA

Scores are in median (first-third quartile); Significant differences between treatment effects are in bold. MOCA, Montreal Cognitive Assessment; RPM, Raven’s Progressive Matrices; ROCF.C, Rey–Osterrieth Complex Figure—Copy condition; ROCF.I, Rey–Osterrieth Complex Figure—Immediate recall condition; ROCF.D, Rey–Osterrieth Complex Figure—Delayed recall condition; FAB, Frontal Assessment Battery; ADL, Activities of Daily Living; IADL, Instrumental Activities of Daily Living; NA, not applicable.

**Figure 3:**
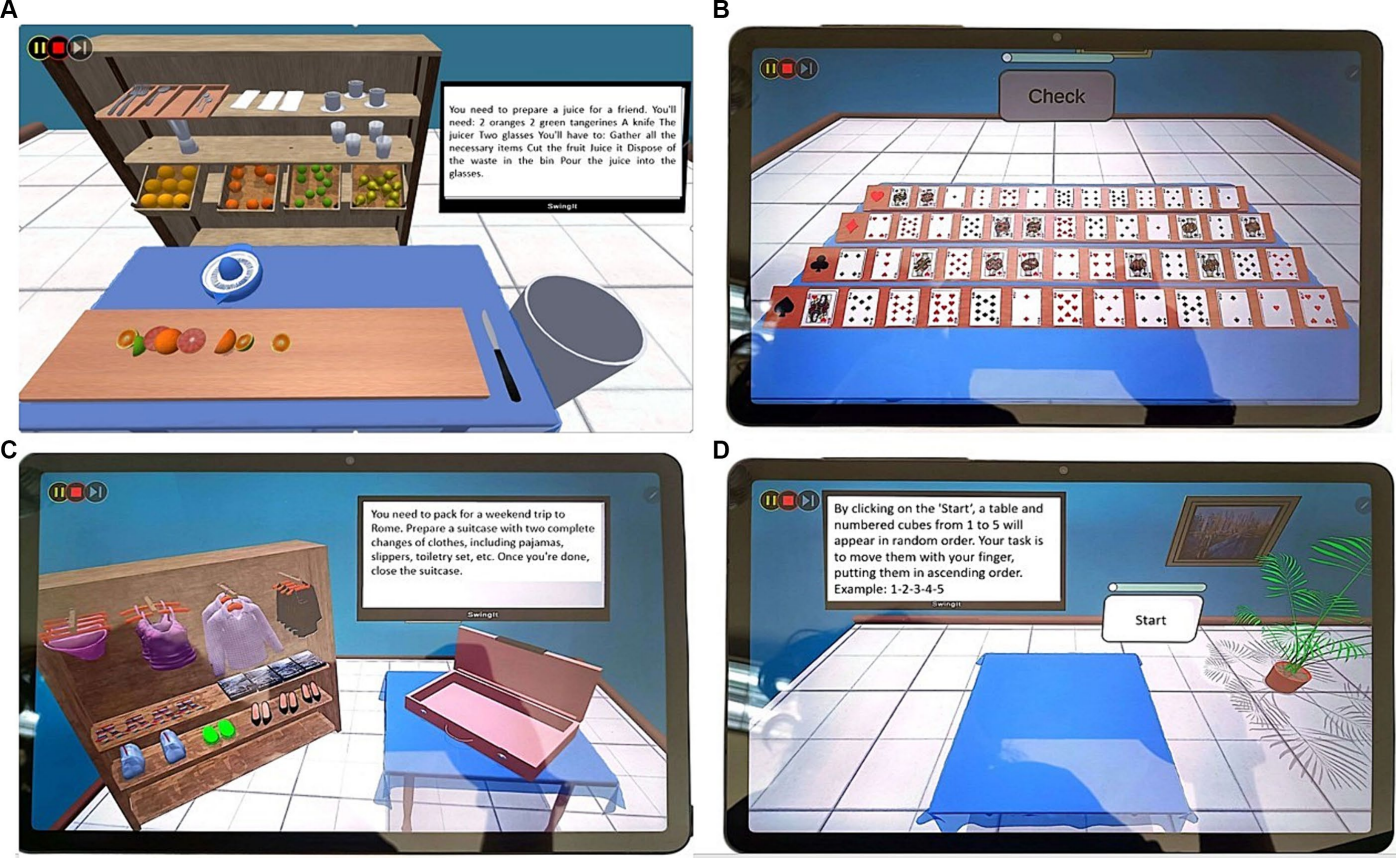
Example of cognitive and daily living tasks proposed to participants. **(A)** AD (daily living) preparation of juice; **(B)** COG (logical deductive reasoning); **(C)** AD (daily living) organizing a trip; **(D)** ADK (visuospatial abilities) arrangement of cubes in ascending order. Screenshots reproduced with permission from Salvatore M. Pappalardo.

## 4 Discussion

The purpose of this preliminary study was to hypothesize the role of the VESPA 2.0 System in MCI to demonstrate how a targeted cognitive rehabilitation process, particularly centered on ecological rehabilitation, leads to recovery of cognitive function and enhances residual cognitive resources for better management of the MCI condition. In recent years, several VR systems have been developed for the rehabilitation of MCI (Zhang et al., 2021). Most randomized controlled trials of VR-based cognitive rehabilitation focus on specific cognitive domains, such as memory and attention, and executive functions (Grazia et al., 2019). Very often, exercises were proposed with abstract elements and with a design very similar to the test used for the assessment (Bondi and Smith, 2014; Portet et al., 2006). Much has been described on the virtual reality system called VRRS used on different neurodegenerative disease and stroke patients, in which there are cognitive exercises that resume, for example, visual search tasks, or categorization based on abstract elements (geometric figures, rulers, and selection of specific targets such as numbers or letters) (Jak et al., 2009; Hung et al., 2020; Formica et al., 2023). VESPA 2.0 system was instead developed for the rehabilitation of multiple cognitive domains requiring the execution of daily living activities at progressive levels literature interest was referred to virtual reality allowed personalized and ecological training of IADL, as Hung demonstrated in other study (Hung et al., 2020). A non-immersive computerized IADL training improved memory, attention, and executive functions in older subjects with MCI, concluding that virtual reality IADL training combined with physical exercise in and outside virtual reality led to similar outcomes, with immersive virtual reality (Formica et al., 2023; Faria et al., 2016). Another study demonstrated the effectiveness of a VR environment in the rehabilitation of cognitive decline. Formica et al. (2023) suggested that Computer-Assisted Rehabilitation Environment (CAREN) training may be effective in the cognitive and emotional domains, improving executive function, anxiety, and depressive symptoms (Tabert et al., 2006). Faria et al. (Oliveira et al., 2021) compared a VR-based intervention that simulated activities of daily living on stroke patients (targeting attention and memory), with conventional rehabilitation. This demonstrates that a cognitive treatment based on ecological rehabilitation improves cognitive performance by using an integrated approach without necessarily having to perform specific and selective training for a single cognitive domain. The use of ecological rehabilitation has also shown great success in patients with overt Alzheimer’s Disease (AD; Piau et al., 2019). This type of treatment, while in AD, has the aim of maintaining daily autonomies, in the patient with MCI, when the functional independence is still preserved; this treatment is provided to enhance these capacities in the early stage of the disease. In particular, this study showed that by working on exercises focused on maintaining ADLs and procedural memory, we improve all those cognitive functions that deteriorate over time. In addition, interaction with our system reported high levels of engagement and motivation, which is important for the improvement of treatment adherence. The good usability and satisfaction scores obtained with the SUS confirmed these observations (Figure 2). Based on this study, VESPA 2.0 is a feasible tool for cognitive rehabilitation, but the efficacy of the tool needs to be demonstrated through adequate experimental design. The limits of our study are certainly the absence of a control group that underwent traditional rehabilitation. In addition, it should consider employing additional methodologies or frameworks to better substantiate feasibility and usability assessments. Relying solely on the System Usability Scale (SUS) is insufficient for a comprehensive feasibility and usability study.

## 5 Conclusion

This study examined the feasibility of VESPA 2.0 in patients with MCI. Overall, the results of this study revealed that cognitive rehabilitation, proposed through an environmentally sound VR system, can be more feasible and have greater usability. However, there are some limitations. First, the absence of a randomized control group might impact the ability to establish causal relationships between the variables considered and the observed outcomes. However, our aim was not to investigate the efficacy of our innovative pathway but rather its feasibility and the potential beneficial role in using simulation’s ADL rehabilitation in MCI patients. Further larger sample studies with a control group receiving conventional therapy are needed to assess the efficacy of our promising protocol. Second, the sample size needs to be larger. Third, multicentric studies should be conducted to evaluate the efficacy of this tool. Our results contribute with new evidence and provide a further understanding of the impact of using simulations of ADLs in the rehabilitation of cognitive deficits. Another strong point of our study is that rehabilitation of the MCI patient on an outpatient procedure seems to be less used in clinical practice. These studies are encouraging for possible inclusion of this therapy in the hospital setting and/or day hospital rehabilitation.

## Data Availability

The raw data supporting the conclusions of this article will be made available by the authors, without undue reservation.

## Footnotes

^1^ http://goo.gl/XCdhLv

^2^ https://zenodo.org/records/10477772
